# ^1^H-NMR Lipidomics, Comparing Fatty Acids and Lipids in Cow, Goat, Almond, Cashew, Soy, and Coconut Milk Using NMR and Mass Spectrometry

**DOI:** 10.3390/metabo15020110

**Published:** 2025-02-08

**Authors:** Brianna Williams, Shamika P. W. R. Hewage, Denzel Alexander, Harshica Fernando

**Affiliations:** Department of Chemistry, Prairie View A&M University, Prairie View, TX 77446, USA; bwillbio@gmail.com (B.W.); rwishvajith@pvamu.edu (S.P.W.R.H.); denale123@gmail.com (D.A.)

**Keywords:** animal milk, plant milk, lipids, fatty acids, fats, phospholipids

## Abstract

**Background/Objectives:** Lipids are an important component of human nutrition. Conventional milk is obtained from animals, and dairy milk is consumed by many people worldwide. Recently, milk consumers have been increasingly shifting towards plant-based milk options. The aim of the study was the qualitative identification of lipid metabolites in animal- and plant-based milk, the identification and comparison of the fatty acids (FAs) of milk, and the qualitative identification of the lipid groups among the milk varieties. **Methods:** Milk samples were obtained from local grocery stores. Lipids were extracted using a modified Folch method and analyzed using nuclear magnetic resonance (NMR) metabolomics. Gas and liquid chromatography mass spectrometry methods (GC-MS and LC-MS) were used to identify the FAs and lipid groups. Lipid weights were compared and the NMR profiles of the lipids analyzed by multivariate statistical analysis. Principal component analysis was performed for the milk lipids obtained from the animal, and plant milk varieties. **Results:** Clustering of NMR data showed two main clusters: cow/almond/cashew and goat/soy/coconut. GC-MS analysis of the methylated fatty acids (FAs) showed the presence of 12:0, 14:0, 16:0, 16:1, 17:0, 18:0, 18:1, 18:2, 20:1, and 20:2 in all milk types, while FAs 19:0 and 20:4 were observed only in the dairy milk. LC-MS data showed common masses that may indicate the presence of mono- and diacyl glycerols and several lysophospholipids among the different types of milk. **Conclusions:** This study shows the advantage of using NMR, GC-MS, and LC-MS to differentiate the lipids among different milk types and compare them on one platform.

## 1. Introduction

Milk and milk products are essential to the human diet in nearly everyone, ranging from infants to adults, and are good lipid sources. The type and amount of fats present in milk may contribute to human well-being. Cow (CW) and goat (GO) milk are the two most common animal milk available for human consumption, while the most commonly consumed plant-based milk beverages in the market are coconut (CO), soy (SO), and almond (AL) milk. Other types, such as cashew milk (CA), oat milk, flaxseed milk, walnut milk, and rice milk, have also emerged in consumer use. Consumers in North America and Europe are increasingly shifting toward plant-based milk options [1,2]. A recent study indicated that dairy milk sales decreased by an average of $456 million/year in the US during the 2017–2019 period, while the sales of other types of milk increased by an average of $123 million/year [1]. The preference for one type of milk over the other is mostly associated with taste, milk allergies, digestive problems, metabolic syndrome, vegan diets, growth hormones, calorie count, cholesterol amount, and other health issues. Plant-based milk lack some of the key ingredients present in natural mammalian milk [3]. Research on milk and its interaction with the human body has been carried out for many years, but there are still many mysteries that need to be solved [4,5,6]. Lipids are among the most important caloric nutrients in milk [6] and vary depending on the type of milk. In the human body, lipids play an important role in energy storage, cellular structure, and signaling. Lipids consist mainly of sterols, triglycerides, free/esterified fatty acids (FAs), and phospholipids (PLs), and are essential for maintaining cellular membranes and other biological roles. Previous data suggest that even though the fat content in milk is ~3–5 wt % [5], it contains several hundred lipid species that play vital roles in human health and nutrition. Hence, lipids are among the most complex materials in nature in regard to their composition [7]. Of the lipids, PLs are gaining increasing attention because of their beneficial effects on human health [3,8]. Recently, there has been an increase in the awareness of the role of FAs and essential FAs (EFAs) in human health and disease prevention, including cardiovascular morbidity and mortality, infant development, cancer prevention, arthritis, hypertension, diabetes mellitus, and neurological disorders [9]. Until recently, the majority of the research work was carried out on CW milk, which has resulted in the discovery of many lipid species. Current characterization of milk lipids using gas chromatography mass spectrometry (GC-MS) [5] involves the profiling of global fatty acid composition, while liquid chromatography mass spectrometry (LC-MS) [5,10] is used to quantify and characterize the physicochemical properties as well as to obtain information about the structure of the lipid species. Herein, the type of column used for the separation can give different types of information. In addition, nuclear magnetic resonance (NMR) spectroscopy has been used for the qualitative/quantitative identification of lipids [8,11]. Lagutin et al., [3] carried out a comprehensive analysis of the lipids present in human, CW, sheep, GO, buffalo, camel, and red deer milk using high performance liquid chromatography (HPLC)-MS, GC, and NMR methods. They performed fatty acid analysis using a GC instrument, and the PLs were analyzed using phosphorus NMR. Blasi et al., analyzed the triacylglycerols in CO, Al, SO, and CW milk using LC-MS [2]. However, with the growing use of plant-based milk, the comparison, identification, and characterization of lipids, including the types of FAs, in the various milk types and defining their usefulness to human health and nutrition will be a challenging task that requires more research.

Metabolomics is a powerful technology that allows us to identify and differentiate low-molecular-weight compounds. NMR and MS based metabolomic techniques have been used in many areas of sciences [3,4,5,6,7,8,9,10,11,12], such as disease identification, the comparison of natural drugs with synthetic drugs, and the identification of metabolic differences in plasma and other tissues [2,12]. Lipidomics, a derivative method of metabolomics, can be utilized to identify the influences of lipids on biochemical functions, such as those of the human genome, diet, microbiome, and other functions of the human lifestyle. These methods are vital foundations for precision medicine tailored to individual biochemical functions and to the investigation of lipids’ cellular pathways in biological systems. Therefore, the identification, differentiation, quantification, and comparison of the types of lipids present in different types of food items are essential. Of the methods that are available for metabolomic analysis [12], ^1^H NMR provides many advantages over the other two methods as a non-destructive and non-targeted technique with minimal sample preparation, and it is capable of detecting all hydrogen-containing molecules in one attempt. The objectives of this work are the qualitative identification of lipid metabolites in animal and plant-based milks using NMR spectroscopy and the identification and comparison of the FAs in the various types of milk using GC-MS and LC-MS to qualitatively identify the lipid groups as differential distributions of lipids based on the type of milk, as milk plays an important role in the etiopathogenesis of many diseases.

## 2. Materials and Methods

### 2.1. Samples and Solvents

Tert methyl butyl ether (MTBE) 99.8%, methyl alcohol 100.0%, hexane, and acetonitrile (ACN) hydrogen chloride solution (3M in methanol) were purchased from the Sigma-Aldrich chemical company (St. Louis, MO, USA). Deionized water was purchased from Mallinckrodt (Blanchardstown, Dublin 15, Ireland). All solvents/reagents and chemicals were of HPLC grade. CW (Borden Vitamin D Whole Milk Grade A Pasteurized), GO (Meyenberg Goat Milk), AL (HEB Almond Milk), CO (SO Delicious Dairy Free Coconut Milk), SO (Silk Original Soy Milk) and CA (Silk Cashew Milk) milk samples were obtained from different local store locations (6 samples for each milk) in Texas, USA.

### 2.2. Extraction of Lipids from Milk and NMR Analysis

Lipids from 6 samples of CW, GO, SO, CO, CA, and AL milk were extracted using a modified Folch extraction method. In each case, 1.0 mL of milk was mixed with 1.5 mL of methanol, 5.0 mL of MTBE, and 500 μL of water, and the mixture was vortexed using the Fisher Vortex Genie 2 (Fisher Scientific, Hampton, NH, USA) for 30 s at room temperature (22–23 °C) as described previously [13,14,15]. Then, 750 μL of water was added, and the mixture was vortexed for two minutes. The samples were then centrifuged at 4000 rpm using the Unico-PowerSpin LXt (Unico, Dayton, NJ, USA) centrifuged for 15 min. From the two layers formed, the top organic layer was removed into a pre-weighed tube. A second extraction was performed using the same volume ratio of solvents and centrifuged to collect the organic layer. The combined organic layers were dried in a water bath (40 °C) with a constant stream of nitrogen to a constant dry weight. The tubes were weighed again and the difference calculated in order to identify the dry lipid weight. The dried samples were stored at −20 °C until data collection.

### 2.3. NMR Data Collection and Analysis

For the NMR experiments, the dried residue was dissolved in deuterated chloroform containing tetramethylsilane (TMS) as an internal standard (Sigma-Aldrich, Milwaukee, WI, USA) [14,15,16]. The final volume was made up to 600 µL in a 5 mm NMR tube (Wilmad Lab glass, Vineland, NJ, USA). ^1^H-NMR spectra were obtained using a Bruker 500 MHz NMR spectrometer with a Bruker Avance Neo console and an Oxford magnet (automated tuning 5 mm 1H/13C/15N cold probe and a 24-position sample case, sample changer, Bruker Inc., San Jose, CA, USA). The spectra were manually phased, and the proton signals were referenced to TMS set at σ = 0.00 ppm. The collected NMR spectra were binned using a General NMR Analysis Toolbox [17]. Binning resulted in a total of 562 data points, and these bin points were subjected to statistical analysis to obtain further information about the lipid profiles. The chemical shifts of clearly identifiable peaks were assigned to various lipids by comparing the published values on lipids, as described previously [14,15,16].

### 2.4. Multivariate Statistical Analysis of NMR Data

The free online web tool Metaboanalyst 5.0 was used for multivariate analysis of the NMR data [18,19,20]. The binned spectra were subjected to unsupervised principal component analysis (PCA) and partial least square discriminant analysis (PLS-DA), which is a supervised method used to observe separations and lipidomic changes between groups. As described previously [20], unsupervised PCA was employed for the initial grouping trend within the dataset and outlier detection. The PLS-DA model denotes the importance of each metabolite in the classification of the study groups in the form of variable importance in projection (VIP). VIP is defined as the weighted sum of squares of the PLS loadings considered the amount of explained Y-variation in each dimension. Metabolites having a score of >1 were considered to be indicative of the discriminatory relevance of the metabolite features. Hierarchical clustering (HC) of the data was performed using the top bin points and the t-test/ANOVA and the PLS-DA VIP models available in the Metaboanalyst 5.0 software program to observe the heat maps of the lipid data. The heatmaps provide intuitive visualization of the data table and help to identify unusually high/low samples/features. The PCA analysis and clustering’s were performed between the two animal milk (CW and GO), four plant-based milk (AL, SO, CO, and CA), and finally between all six milk varieties (CW, GO, AL, SO, CO, and CA).

### 2.5. Transesterification and Preparation of the Lipid Samples for Fatty Acid Analysis

Tert methyl butyl ether and hydrogen chloride solution were added to the dry lipids in sample vials, which were sealed with tight caps and vortexed using the Fisher Vortex Genie 2. The sealed vials were placed on a hot plate for one hour at 200 °C. After one hour, the samples were cooled, and 1.0 mL of HPLC grade hexane was added using a syringe. The sample was vortexed again and kept at room temperature for 1 h. Removed 50 μL of upper phase and added to a 1.5 mL GC vial. The removed upper phase was mixed with 450 μL HPLC-grade hexane. The vial was sealed and used for GC-MS analysis.

### 2.6. Gas Chromatography Mass Spectrometry (GC-MS) of the Fatty Acid Methyl Esters

A GC/MS (Agilent 7890A/5975C) with a DB-5MS column (30 m × 0.25 mm × 0.25 µm, Agilent p/n 122-5532) was used in the fatty acid methyl ester analysis. The instrument performance on each day was monitored by running a daily tune file. Total flow was set at 1 mL/min helium. Oven temperature was set at 280 °C, and each sample was run for a total of 59 min. Samples from each milk variety were run to observe the fatty acids present in each type of milk. In an attempt to observe and identify the fatty acids present, the standard mixture of 37 fatty acid methyl esters (Sigma Aldrich, CRM 47885) was used to identify the methylated fatty acids and match them to the reference sample in retention time and mass. In addition, all significant peaks were matched using the MassHunter software program, which was available in the instrument. The methylated fatty acids with a score above 95% were taken, and compared among the different milk varieties.

### 2.7. Liquid Chromatography Mass Spectrometry (LC-MS) of the Lipids

LC-MS analysis of the milk lipids was carried out using an Agilent 1200 LC system in conjunction with an Agilent 6130 quadrupole LC/MS and two different columns. All lipids were dissolved in 1.5 mL of an MTBE and methanol (1:1) mixture. The autosampler was used to inject 5 μL of this mixture. In the first analysis, a Varian C18 column (150 × 4.6 mm) was used with a binary solvent system: solvent A—ACN: water (60:40), 10 mm ammonium acetate, and solvent B—ACN (90:10), 10 mm ammonium acetate—a method similar to the work by [8,21] with minor modifications. The flow rate was 0. 25 mL/min and the experiment was carried out using the following gradient: 10% A at 0 min, 10% A at 0.2 min, 5% A at 5.2 min, 5% A at 8.9 min, 0% A at 15.4 min, 10% A at 15.4 min, 10% A at 15.6 min, and 10% A at 18 min, with a total run time of 20 min. The system was set to function in negative ionization mode with a mass range of *m*/*z* between 300 and 1200. The spray voltage was 3000 V, the capillary temperature was kept at 320 °C, and the heater temperature was set at 300 °C. In the second analysis, an Agilent HILIC column (100 × 2.7 mm) was used and followed the method of [22] with minor modifications. The mobile phase consisted of 0.1% ammonium acetate in ACN (A) and 0.1% ammonium acetate in methanol (B), with the following gradient elution profile: 100% A at 0 min, 90% A at 0.1 min, 80% A at 5.0 min, 75% A at 25 min, and 100% A at 27 min, with a total run time of 30 min. The flow rate was set at 0.4 mL/min. The mass spectrometer operated in negative ion electrospray ionization mode with a scan range of *m*/*z* 200–1200. In both separation methods, the column temperature was maintained at 27 °C. The obtained masses were compared with the LIPIDMAPs database to identify the various lipid groups. Three samples were analyzed for each milk variety, and the masses reported here are the masses that were detected in all three samples.

## 3. Results

### 3.1. Milk Composition, and Lipid Weights

**Figure 1:**
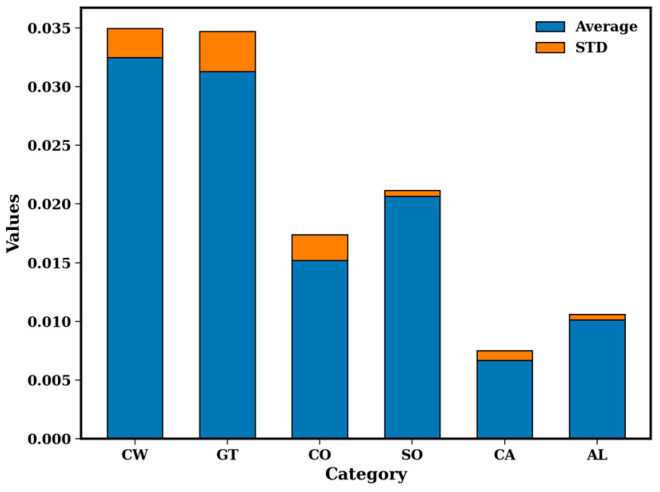
Comparison of lipid weights obtained after extraction of the lipids. The data reveal the average of five samples.

Table 1 shows the milk compositions listed on the milk cartons. The calorie content, expressed as the number of calories present in one cup, is in the order CW > GO > SO > AL > CO > CA. However, the information given shows that the total fat content changes in the order CW > GO > SO = CO > AL > CA, while the saturated fat content changes in the order CW > GO = CO > SO (AL and CA contain no saturated fat). Of the total fat, none of the milk varieties contain any trans fats. Information regarding the presence of monounsaturated fat and polyunsaturated fat was not available for all the milk types considered. From the information provided, SO and GO milk have the highest polyunsaturated fat content, while AL milk has the highest monounsaturated fat content. Cashew milk is not as popular as AL, SO, or CO but does not contain any saturated and polyunsaturated fats and has the lowest total fat content. Hence, it is interesting to compare the lipid pattern of CA with that of the other plant milk varieties. Both animal milk varieties contain 35 and 25 mg of cholesterol, as compared to the plant-based beverages. Fat contains several hundred lipid species, which play vital roles in human health and nutrition, so knowing and understanding them are important. When the milk lipids were extracted, the weights were recorded prior to any analysis using the NMR, GC-MS, or LC-MS instruments. Figure 1 shows the distribution of the average lipid weights obtained after extraction. The data indicate that the lipid weights are highest in CW and lowest in CA, with the order varying as CW> GT > SO > CO > AL > CA. The animal milk have higher weights, consistent with the caloric values reported in Table 1.

### 3.2. ^1^H NMR of Lipids and Comparison of Lipid Profiles

**Figure 2:**
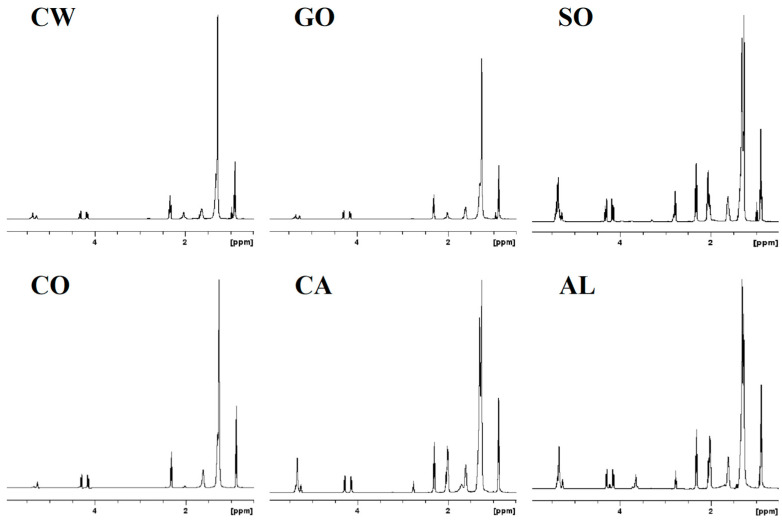
Representative one-dimensional ^1^H NMR spectra of lipid extracts from CW, GO, SO, CO, CA, and AL milk using a Bruker 500 MHz NMR spectrometer.

**Figure 3:**
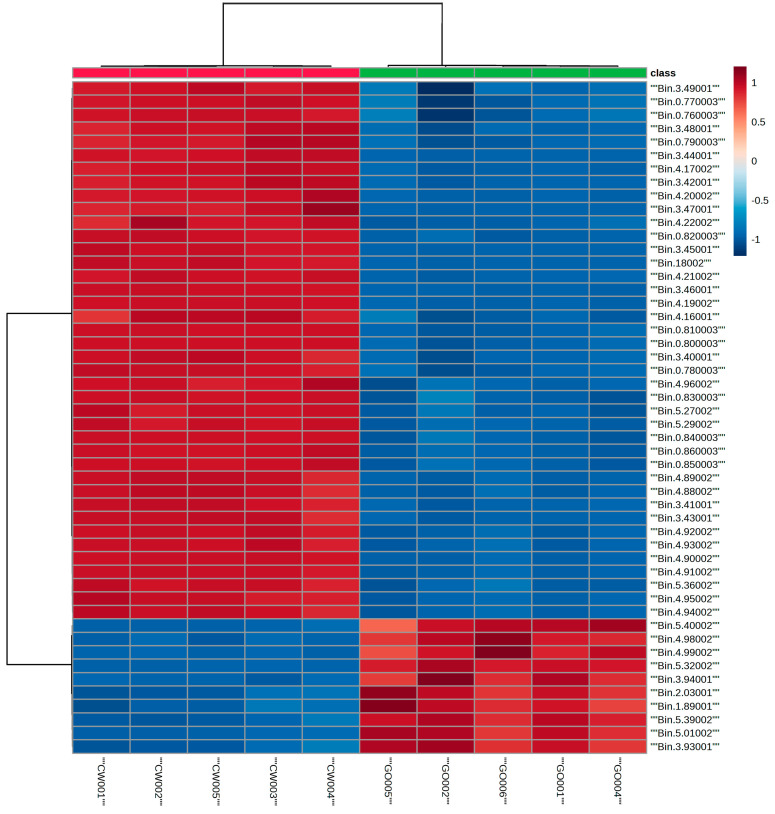

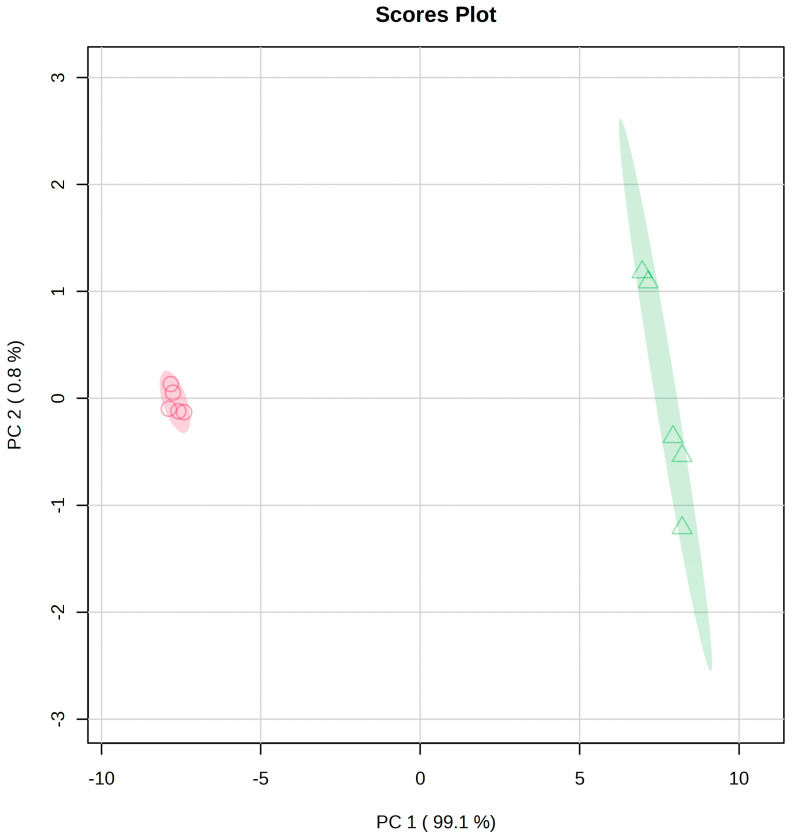
Heat maps and PCA analysis of ^1^H-NMR data of CW (red circle) and GO (green triangle) milk lipids. (**top**) represents the HC analysis, and (**bottom**) represents the PCA analysis. Herein, each point represents a value calculated from an individual spectrum.

**Figure 4:**
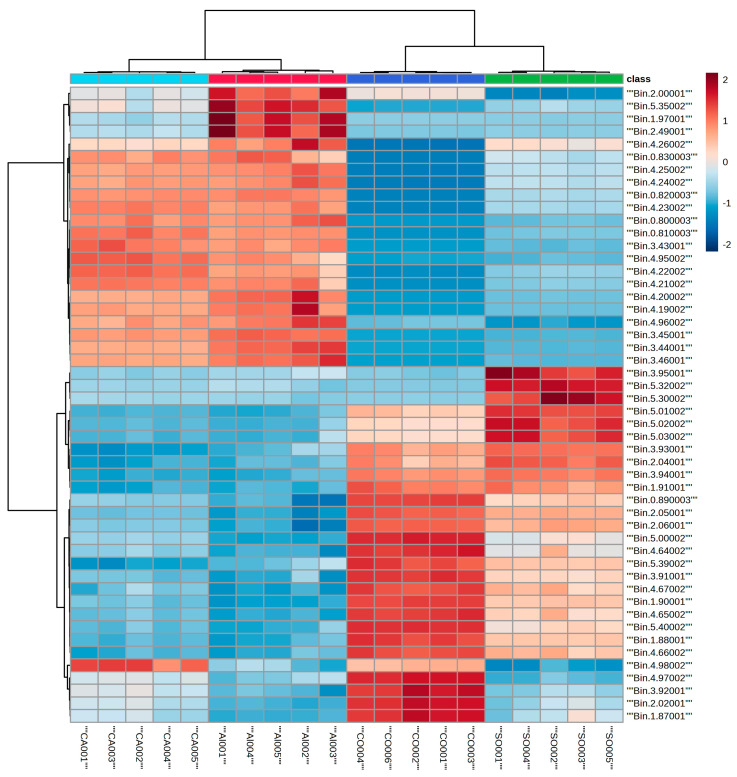

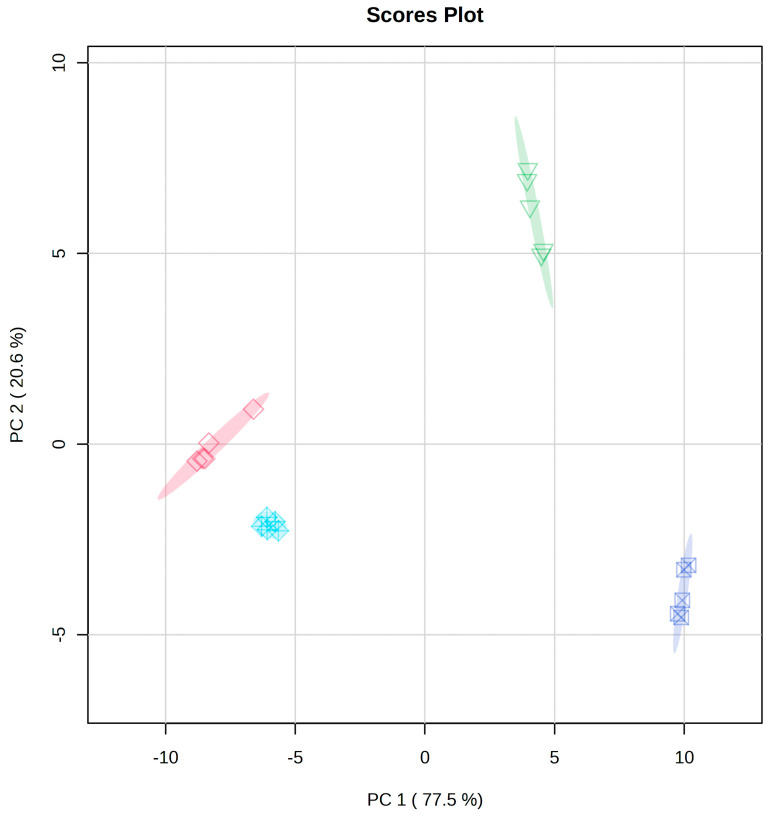
HC (**top**) and PCA (**bottom**) analyses of the ^1^H-NMR spectral data of lipids of AL (twisted square, red), SO (inverted triangle, green), CO (open crossed square, dark blue), and CA (filled twisted crossed square, light blue). The two-dimensional plot of the data shows a clear separation of SO from the other types of plant-based milk.

**Figure 5:**
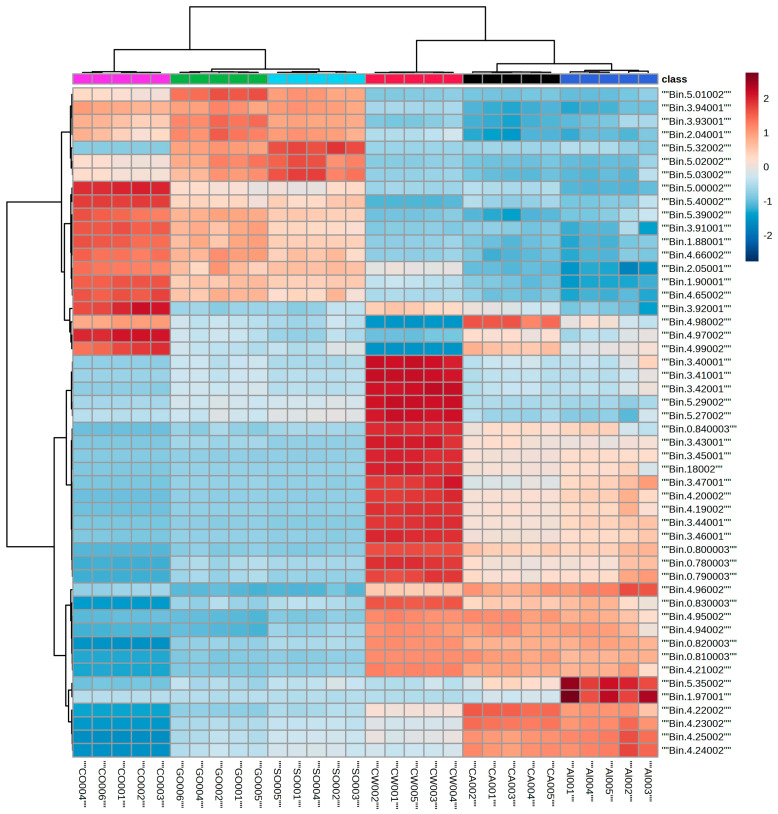

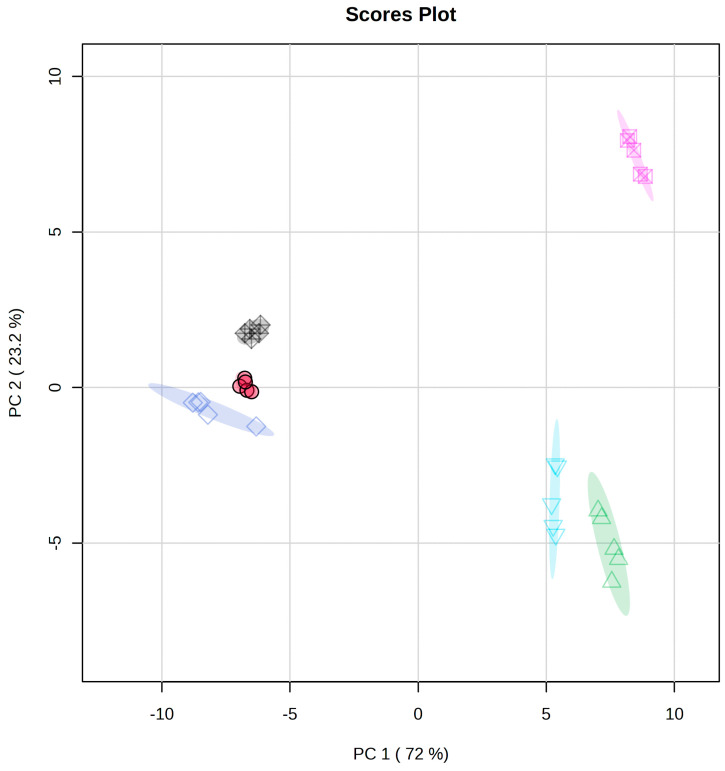
HC (**top**) and PCA (**bottom**) analyses of ^1^H-NMR spectral data of lipids of all milk varieties. The symbols (CW (red circle), GO (green triangle), AL (twisted square, dark blue, SO (inverted triangle, light blue), CO (open crossed square, pink), and CA (filled twisted crossed square, black) in the PCA figure are the same as in Figure 4 and Figure 5. The two-dimensional plot of the PCA data shows three main clusters: CA, CW, and AL are in one cluster and SO and GO are in another, while CO stands alone.

To better understand the variations and compatibilities of the different types of milk, we employed proton NMR spectroscopy in conjunction with multivariate statistical analysis to identify the altered lipids. Figure 2 shows the ^1^H NMR lipid profiles obtained for the six different milk varieties. In the profiles, the ^1^H NMR signals corresponding to cholesterol, fatty acid chains (saturated and unsaturated), the glycerol backbone, and phospholipids are observed. Visual inspection of the NMR profiles shows differences among the milk types. To better understand the differences in the NMR profiles, clustering of the NMR data was performed using both HC and PCA methods, and the corresponding profiles are shown in Figure 3, Figure 4 and Figure 5. The two-dimensional HC plot of the data shows a clear separation of the CW and GO lipids.

In the PCA analysis, the lipid data showed two significant principal components, which accounted for 99.9% of the total variance in the dataset. Principal component 1 corresponds to 99.1%, and component 2 corresponds to 0.8%. The main differences were observed in the regions of 0.76–0.78, 0.80–0.86, 3.40–3.49, 4.16–4.21, 4.90–4.96, and 5.27–5.29 ppm, as observed by the HC profiles. These signals correspond to the presence of CH_3_ groups, free cholesterol, and triglycerides in the extracted lipids. Figure 4 shows the HC and PCA analyses of the plant-based milk varieties. PCA analysis of the plant lipid data showed two significant principal components, which accounted for 98.1% in the total variance of the dataset. The data for component 1 corresponds to 77.5%, while that for component 2 corresponds to 20.6%.

Figure 4 indicates a clear separation of the four types of milk, with CO and SO milk well separated from AL and CA milk. This separation is clearly demonstrated in the HC clustering pattern, with CO and SO clustered together as compared to CA and AL. The regions of interest among the two major clusters correspond to the NMR regions corresponding to 0.81–0.83, 1.87–1.90, 2.02–2.06, 3.43–3.46, 3.91–3.95, 4.19–4.16, 4.65–4.67, 4.95–4.96, 4.97–5.03, 5.30–5.32, and 5.39–5.40. Among the changes, some ppm values were upregulated while the others were downregulated. Areas of interest were the changes observed among the triglycerides, phospholipids, and unsaturation’s. Figure 5 shows the HC and PCA analyses of all six milk types. In the PCA analysis, all the lipid data showed two significant components and accounted for 95.2% of the total variance of the dataset, with the first component corresponding to 72% and the second component corresponding to 23.2%. The data show similarities among some types of milk. CW, AL, and CA were close to each other, while SO and GO were away from that cluster (but showing similarities), and CO was away from the other five types of milk.

The HC analysis of the six milk varieties can be grouped into two main groups: AL, CA, and CW in one group, and CO, SO, and GO in the other group. The significant bins associated with the groups are 0.78–0.84, 2.04–2.06, 3.44–3.46, 3.91–3.94, 4.19–4.25, 4.65–4.66, 4.94–4.96, 4.97–5.03, 5.27–5.29, and 5.38–5.40. The differences observed using the ^1^H NMR analysis are very qualitative, showing elevated levels in different regions for the ^1^H NMR signals corresponding to the PL backbone, saturation/unsaturation, cholesterol peaks, different alkyl chains, and triglycerides. Of the lipids, PLs are gaining increasing attention because they are known to have an effect on alcoholic and nonalcoholic fatty liver diseases [14,15,16,23]. In terms of groupings, this analysis helps us to better understand where each milk may be grouped in terms of its lipid content.

### 3.3. Fatty Acid (FA) Identification and Comparison Among the Different Milk Varieties

Fatty acids play a significant role among the lipids and are an essential component of lipids in animals, plants, and microorganisms. To understand the FAs present, a qualitative analysis of FAs was carried out. Identification of the FAs was performed by esterification of the carboxylic acid group and through GC methods. As FAs are primarily in the form of esters (triglycerides, phospholipids, and cholesteryl ester), hydrolysis was performed prior to the methylation. Table 2 lists the FAs present in the different types of milk. The FAs observed in this study were matched with previous studies and are indicated in the table [3,24,25,26,27,28,29,30,31,32,33,34]. The results in Table 2 show that some FAs are present in all milk types (12:0, 14:0, 16:0, 16:1, 17:0, 18:0, 18:1, 18:2, 20:0, and 20:1), while 8:0, and 10:0 were present in CW, GO, CO, and AL milk. FAs with 6:0 were detected in the CW, GO, and CO samples. We did not observe 4:0 FA in any of the samples analyzed. This may be due to the limitations of our system. The odd-chain FAs, with chains of 11:0, 13:0, and 15:0, were present in CW, GO, and CO milk types, while 19:0 and 22:5 FAs were found only in CW and GO milk, and 15:0 FAs were also detected in AL and SO milk. Both animal milks contained 20:4 and 22:5 FAs, but 20:3 and 22:4 FAs were found only in CW milk. FA 21:0 was found in GO and SO milk.

### 3.4. Lipid Group Identification Using LC-MS

To better understand the lipid groups and the attachment of different FA chains to the various classes of lipids, this study utilized a LC-MS method with two distinct columns consisting of different stationary phases. Using the C18 column, many different lipid classes can be identified, while the HILIC column enables the specific analysis of only the polar lipids. Table 3 and Table 4 list the *m*/*z* values of the lipids obtained using the two columns. Some lipid masses were common among different milk types.

In the analysis using the C18 column, *m*/*z* values of 313 (20:0 lysophosphatidiyl inositol- (LPI) [M-2H]^2−^), 314 (18:1 spingoid bases [M-H]^−^), 465 (20:0 lysophospatidic acid (LPA) [M-H]^−^), and 607 (35:1 diacyl glycerols (DG) [M-H]^1−^), were common for all milk types. *The m*/*z* value of 537 (20:1 lysophosphatidiyl glycerol (LPG) [M-H]^−^) was absent in CO. *m*/*z* values of 466 (LPA14:0 lysophosphatidiyl choline (LPC), 17:0 lysophosphatidiyl ethanol amine (LPE), and 16:0 (PE all [M-H]^−^) were present in Al, CA, GO, and SO, *m*/*z* 437 (18:0 LPA [M-H]^−^) in AL, CA, CO, and GO, *m*/*z* 411 (PI 33:0 [M-2H]^2−^) in AL, CA, GO, and SO, *m*/*z* 345 (18:1 [M-H]^−^ and/or 16:1 ([M + OAc]^−^ FA esters), and 467 (16:1 LPA [M + OAc]^−^) in AL, CO, GO, and SO, while *m*/*z* 353 (12:0 LPA, 18:2 monoglycerol (MG) with [M-H]^1−^) was common to Al, CO, and SO. The scan *m*/*z* ranges for the C-18 and HILIC columns were 200–1000 and 350–1200, respectively, and the scan ranges were selected based on the properties of the column. The *m*/*z* values obtained for the HILIC column showed that *m*/*z* value of 387 (20:0 (MG) [M-H]^−^ or 36:0 PC/39:0 PE [M-2H]^2−^) was common to all milk types, and *m*/*z* values of 469 (16:0 LPA [M + OAc]^−^) and 551 (31:1 DG [M-H]^−^) were common to all except CO, while *m*/*z* 633 (31:0 PA [M-H]^−^) was common to only CA, CO, CW, and SO. A *m*/*z* value of 685 (20:1 LPI [M + OAc]^−^ and 35:2 PA [M-H]^−^) was common to CA, CO, and SO, while *m*/*z* 1003 (36:0 ceramide phosphoinositols (MIPC) [M + OAc]^−^) and 1043 (40:0 MIPC [M-H]^−^) or 34:0 MIPC [M + OAc]^−^) were common to AL, CA, and SO, and *m*/*z* 415 34:3 PI [M-2H]^2−^) was common to CW, GO, and SO. In our analysis, we attempted to find common masses among the different milk types and then to validate the presence of the lipid group or the fatty acid chain using the lipid maps database. In the absence of many lipid standards and MS/MS analysis, our data give a glimpse of the FA chains binding to different lipid groups. In the HILIC column data analysis, we selected only the polar lipids/glycerols to match the observed *m*/*z* values, while the data from the C18 column were compared with the *m*/*z* values of cholesterol esters, fatty acid esters, glycerol lipids, and glycerophospholipids.

## 4. Discussion

Milk is sold on the basis of fat/calorie content and the main milk lipids are triglycerides [3,5]. Other lipids present in milk are diacylglycerol, monoacylglycerols, PLs, cholesterol (free and esterified), and free FAs, all of which have different activities in the human body [3,5]. Milk from different sources vary significantly in their lipid profiles and can be related to the degree of unsaturation of fatty acids [8,10]. As the lipid matrix is very complex [3], milk lipids from animal and plant origins were compared in this study using NMR, GC-MS, and LC-MS methods to better understand the correlations (if they exist) between the different milk varieties. Lipid components have different biological functions in the body and need to be monitored when taking milk for consumption [4,9,10]. Hence, having an idea of the comparison of the lipids in the different varieties is important. No other study has provided an NMR-based lipid profile of the lipids analyzed here. Table 1 shows the basic information available on the milk containers that gives consumers a brief explanation of the types of fats available, together with the amounts of calories, vitamins, cholesterol, carbohydrates, and sugar. However, there is no detailed description of the fats present. The availability of advanced instrumentation, coupled with statistical tools, offers researchers the opportunity to analyze these different fat components [3,11,12,22,24]. In studying the lipid profiles, each analytical technique has its own advantages. Many groups have conducted FA, PL, and triglyceride analyses using GC-MS or LC-MS techniques [2,3,5,8,10,24,25,26,27,28,29,30,31,32,33,34]. However, information about all the milk types listed here has not been compared simultaneously. Analyses of FAs are complicated, as they are of high molecular weight and are less volatile. FAs are converted to fatty acid methyl esters to increase volatility [3]. They exist in different forms and are characterized by length, the number of carbon atoms, saturation or unsaturation, and linear or branched configuration(s); they are mainly analyzed using GC-MS methods [3,9,24,25,29,30,32]. The disadvantage of using this method is the need for the lipid extract to be converted to fatty acid methyl esters [35]. Analysis of FAs by NMR alone will not give us much information about the individual FAs, and identification will be challenging. However, identification of the different lipid groups can be carried out using NMR [3]. NMR methods have been used in the identification of the phospholipids [3,10]. However, despite the technique being sensitive, more research needs to be performed to obtain accurate results [10]. LC-MS methods can be used to identify different lipid groups [2,3,8,22,26,27,30,31,36,37], but different types of columns need to be used to get a detailed identification of the lipid groups. By using NMR and statistical methods, a qualitative comparison of the lipid profiles can be obtained without detailed descriptions of the FA chains or the lipid groups [6].

The results of HC and PCA analyses of animal- and plant-based milk and the comparison of all six milk varieties, showed separations and different groupings. These separations were attributed to elevated or decreased levels of the signals corresponding to different ^1^H signals in the lipid groups. In the animal milk comparison, signals corresponding to the presence of different alkyl chains, cholesterol, or glycerol separated the two groups from each other (Figure 3). In the absence of cholesterol in plant-based milk (Table 1), the clustering differences were due to the elevated or decreased levels of the signals corresponding to glycerol, phospholipid, and unsaturation of the fatty acid chains (Figure 4). A comparison of the data in Table 2 shows some FA differences between CW and GO milk as well as among the different plant-based milk varieties studied here. One reason that consumers tend to use plant-based milk options is the presence of cholesterol in animal milk, as cholesterol is linked to cardiovascular diseases [38]. Triglycerides, which are esters and composed of glycerol and fatty acids, are an excellent energy source. An excess of triglycerides can lead to hypertriglyceridemia and is known to correlate with cardiovascular disease [38,39]. Dairy fat contains more than 400 different FAs [35,40].

FAs are important to human health, and understanding the types of FAs present in each type of milk will provide information to consumers. FAs are known to cause liver injury via oxidative stress and inflammatory pathways [41], and saturated fatty acids coincide with high blood cholesterol and heart disease [3,8,9]. The results shown in Figure 5 demonstrate two main clusters with differences associated with alkyl chain distribution and cholesterol-, phospholipid-, and glycerol-related peaks. In the first cluster, AL and CA were grouped with CW milk, while in the second cluster, SO and GO were more closely related but had some similarities with CO. Looking at the clustered fatty acids by themselves will not give a clear understanding, as these FAs were obtained by hydrolysis followed by methylation. Unsaturated protons, phosphatidylcholine, and phosphatidylethanolamine signals contributed to the separation. The absence of *m*/*z* 466 and 469 in CO may have contributed to the separation of the CO group from the other five groups, while GO and SO had several *m*/*z* values that were common between them and may have resulted in the closer grouping. Many *m*/*z* values were common between AL and CA.

Of the FAs obtained in Table 2, the majority have an even number of carbon atoms. However, some FAs possess odd numbers of carbons and are biosynthesized and metabolized slightly differently from their even-chained relatives [40]. Among the FAs, polyunsaturated fats include critical FAs omega-6 and omega-3 FAs [9,42]. Though at a relatively low level, many milk varieties contain these two polyunsaturated FAs. Other essential FAs needed for normal human development are linoleic C18:2 (n-6), α-linolenic C18:3 (n-3), eicosapentaenoic C20:5 (n-3; EPA), docosahexaenoic C22:6 (n-3; DHA), γ-linolenic C18:3 (n-6), and arachidonic C20:4 (n-6) acids [42,43]. As FAs have also been implicated in the prevention of cardiometabolic disease, and many use milk and milk-related products as a source of obtaining FAs [44], comparisons are very useful. Many of the FAs listed in Table 2 were found in independent analyses of the FA chains by others, and most studies have focused on CW and GO milk. However, the presence of 19:0 in GO milk was not listed in the papers describing the analysis of milk FAs. Fernandes at al. reported the presence of 15:0 and 17:1 FA in the analysis of AL oil, and we detected this FA in AL milk [45]. Not many papers exist on the identification of FA in CA milk. The presence of FAs 14:0, 17:1, and 19:0 in CA milk is reported here for the first time; however, FA 14:0 and 17:1 have been detected in the FA analysis of CA raw nuts [46]. Cashew nuts contain high amounts of oleic and linoleic acids [46]. In our analysis, the 18:3 FA was detected only in soy milk. This may be due to the amount that was present in our samples and a limitation in our study. Many other papers have described the presence of 18:3 in other types of milk. In our work, 17:1 FA was absent in CO and present in all the other five types of milk, consistent with previous analysis. Lagutin et al., Maetinez-Padilla et al., and Moore et al., [3,24,30] have performed an extensive analyses of FA milk profiles. Work by Lagutin et al. [3] focused only on dairy animals (New Zealand), while the work by Moore et al., [24] focused on AL, CO, SO, CW, and GO and included rice and oat milk; samples were collected from Italy. Moore et al., [30] collected samples from Denmark and compared many plant milk types with cow’s milk. CA was not compared in these studies, and all samples used in this study were collected from grocery stores in Texas. As milk composition varies depending on type and origin, this study will enhance the existing information about the lipids/FAs in milk lipids.

PLs, another important source of lipids present in milk fats, have also been implicated in alleviating steatosis and in infant gut and brain development and individual PL identifications of these lipid varieties will be more beneficial [10,23,47,48]. In addition, the human body needs cholesterol to build healthy cells, and excess may lead to other diseases [38]. Bovine milk has been part of the human diet for a long time [49,50]. However, the increasing demand for non-dairy products has promoted more investigations into animal- and plant-based products, and this study provides only a qualitative analysis of the milk lipids. This study still has some limitations and requires further improvement in the future. More detailed research needs to be carried out to understand and identify many other lipid groups that can be present among these milk varieties [50,51] and to identify potential biomarkers (metabolites) to detect any authenticities of milk.

## 5. Conclusions

In conclusion, this study provides relationships among the lipids of animal- and plant-based milk and demonstrates that ^1^H NMR lipidomics can be used to obtain different clustering patterns among different food types. Since many lipids exist, the identification of all the different types is a challenging task. This work shows that recent advancements in analytical instrumentation allow us to differentiate the lipid species present in a given sample, helping to establish metabolite analysis, which can be linked to nutritional aspects of milk.

## Figures and Tables

**Table 1:** Comparison of the ingredients present in cow, goat, almond, soy, coconut, and cashew milk.

Ingredients (240 mL/1 cup)	Cow Milk	Goat Milk	Almond Milk	Soy Milk	Coconut Milk	Cashew Milk
Calories	160	140	60	110	45	25
Total Fat (g)	8	7	2.5	4.5	4.5	2
Saturated Fat (g)	5	4	0	0.5	4	0
Trans Fat (g)	0	0	0	0	0	0
Polyunsaturated Fat (g)	n/a	2.5	0.5	2.5	n/a	0
Monounsaturated Fat (g)	n/a	1	1.5	1	n/a	1
Cholesterol (mg)	35	25	0	0	0	0
Sodium (mg)	130	115	150	90	15	160
Vitamin D (mcg)	2.5	3	5	3	n/a	2.5
Iron (mg)	0.1	n/a	0	1.1	n/a	0.5
Total Carbohydrate (g)	12	11	8	9	2	2
Dietary Fiber (g)	0	0	0	2	1	0
Total Sugar (g)	11	11	8	6	0	0
Protein (g)	8	8	1	8	0	<1
Calcium (mg)	300	300	560	470	n/a	450
Potassium (mg)	400	420	250	370	40	0

n/a data not available.

**Table 2:** Fatty acids observed (X) in the milk lipids analyzed by GC-MS.

Fatty Acid Name	CW	GO	CO	AL	SO	CA
Hexanoic acid (6:0)	X [3,24,25,26,27,28]	X [3,24,26,29]	X [24,30]			
Octanoic acid (8:0)	X [3,24,25,26,27,28]	X [3,24,26,29]	X [24,30]	X [24]		
Decanoic acid (10:0)	X [3,24,25,26,27,28]	X [3,24,26,29]	X [24,30,31]	X [24]		
Undecanoic acid (11:0)	X [24,25,26,27]	X [24,26,29]	X [24]			
Dodecanoic acid (12:0)	X [3,24,25,26,27,28]	X [3,24,26,29]	X [24,30,31]	X [24,30]	X [24]	X [34]
Tridecanoic acid (13:0)	X [24,25,26,27]	X [24,26,29]	X [24]			
Tetradecanoic acid (14:0)	X [3,24,25,26,27,28]	X [3,24,26,29]	X [24,30,31]	X [24]	X [24,31,32]	X
9-Tetradecenoic acid (14:1)	X [3,24,25,27,28]	X [3,24,29]				
Pentadecanoic acid (15:0)	X [3,24,25,26,27,28]	X [3,24,26,29]	X	X	X [24,32]	
Hexadecanoic acid (16:0)	X [3,24,25,26,27,28]	X [3,24,26,29]	X [24,30,31]	X [24,30]	X [24,31,32,33]	X [33,34]
9-Hexadecenoic acid (16:1)	X [3,24,25,26,27,28]	X [3,24,26,29]	X [24,30]	X [24,30]	X [24,31,32,33]	X [33,34]
Heptadecanoic acid (17:0)	X [3,24,25,26,27,28]	X [3,24,26,29]	X [24]	X [24]	X [24,32,33]	X [33,34]
cis-10-Heptadecenoic acid (17:1)	X [24,25,26,27,28]	X [24,26,29]		X	X [32]	X
Octadecanoic acid (18:0)	X [3,24,25,26,27,28]	X [3,24,26,29]	X [24,30,31]	X [24,30]	X [24,30,31,32,33]	X [33,34]
8-Octadecenoic acid (18:1)	X [3,24,25,26,27,28]	X [3,24,26,29]	X [24,31]	X [24,30]	X [24,30,31,32,33]	X [33,34]
9,12-Octadecadienoic acid (18:2)	X [3,24,25,26,27,28]	X [3,24,26,29]	X [24,30,31]	X [24,30]	X [24,30,31,32,33]	X [33,34]
9,12,15-Octadecatrienoic acid (18:3)					X [31]	
Nonadecanoic acid (19:0)	X [27]	X				
Nonadecaenoic acid (19:1)	X [27]	X [24]			X [24]	X
Icosanoic acid (20:0)	X [3,24,26,27,28]	X [3,24,26,29]	X [24]	X [24]	X [24,31,32,33]	X [33,34]
9,-Icosenoic acid (20:1)	X [3,26,27]	X [3,24,26,29]	X [24]	X [24]	X [24]	X [34]
Di-cosenoic acid (20:2)					X [24]	
5,8,11 Icosatrienoic acid (20:3)	X [3,24,25,26,27]					
5,8,11,14-tetraenoic acid (20:4)	X [3,24,25,26,27]	X [3,24,26,29]				
Heneicosanoic acid (21:0)		X [24,26]			X	
Docosanoic acid (22:0)	X [24,26]	X [24,26]	X [24]		X [24,31,32,33]	X [33,34]
7,10,13,16-docosatetraenoic acid (22:4)	X [27]					
7,10,13,16,19-docosapentaenoic acid (22:5)	X [3,24,27]	X [3,24]				
Tricosanoic acid (23:0)	X [26,27]				X	
Tetracosanoic acid (24:0)	X [3,26,27]		X [24]		X [24,32,33]	X [33,34]

FA 20:2 was observed only in SO milkfat and FA 24:0 was present in all except GO and AL milk lipids.

**Table 3:** *m*/*z* values obtained using the C-18 column. Numbers common to at least three varieties are highlighted. Each color represents an individual mass.

Milk Type	Mass Readings
AL	313.1, 314.1, 315, 327.2, 345.2, 347.3, 353.2, 354.1, 411.2, 437.3, 465.3, 466.3, 467.2, 485.4, 509.1, 537.3, 538.4, 593.5, 607.3, 609.2, 621.3, 663.5, 803.1, 863.5
CA	309.1, 313.1, 314.1, 339.2, 341.1, 342.2, 357.2, 367.2, 411.2, 433.1, 437.3, 441.1, 465.3, 466.3, 473.3, 504.4, 505.3, 509.4, 535.5, 537.5, 551.5, 591.5, 607.3, 608.4, 609.3, 683.3, 691.3, 705.1, 735.8, 759.9, 762.5, 803.2
CO	313.1, 314.1, 321.2, 345.2, 353.2, 387.3, 404, 423.3, 437.3, 441.2, 454.3, 465.3, 467.4, 493.4, 509.5, 593.5, 607.3, 621.5, 635.3, 683.2, 705.1, 762.5, 803.3
CW	309.1, 313.1, 314.1, 357.2, 358.3, 381.4, 395.3, 431.1, 439.58, 465.3, 466.3, 481.5, 495.5, 524.5, 537.3, 607.3, 665.3, 705.4, 736.4, 746.5, 762.7, 965.5
GO	313.1, 314.1, 315, 327.2, 345.2, 347.3, 353.2, 354.1, 411.2, 437.3, 465.3, 466.3, 467.2, 485.4, 509.1, 537.3, 538.4, 593.5, 607.3, 609.2, 621.3, 663.5, 803.1, 863.5
SO	313.1, 341.1, 342.1, 345.2, 369.2, 381.3, 411.2, 421.1, 431.1, 432.1, 465.3, 467.2, 475.1, 476.1, 481.2, 521.1, 537.3, 571.3, 595.3, 607.3, 635.3, 683.2, 684.3, 685.2, 693.5, 777.6

**Table 4:** *m*/*z* values obtained using the HILIC column. Numbers common to at least three varieties are highlighted. Each color represents an individual mass.

Milk Type	Mass Readings
AL	387, 388, 404.1, 469.1, 511, 551.1, 552, 592.8, 633.2, 635, 674.9, 705.2, 714.9, 717.2, 756.9, 757.2, 799, 839.1, 880.1, 921.2, 962.2, 1003, 1043, 1125.2
CA	387, 388, 397.25, 401.1, 423.1, 453, 469.1, 485, 517.2, 551.1, 552, 580.8, 608.9, 621.3, 633, 634.9, 635.4, 683.1, 685.2, 690.8, 714.9, 731, 773, 774.9, 777.8, 787.1, 799, 838.9, 920.8, 1003, 1043
CO	283.2, 331.1, 341.1, 387, 459, 635.2, 685, 787.1, 944.3
CW	387, 389.3, 391.3, 415.3, 439.2, 457, 469.1, 489, 490.9, 501, 529.3, 551.1, 596.6, 675.8, 704.9, 733, 800
GO	377.1, 387, 389.3, 401.1, 415.3, 431.3, 465.3, 469.1, 537.3, 551.1, 568.5, 581, 598.5, 624.4, 633, 639.2, 662.9, 689.3, 690.8, 715, 715.3
SO	353.1, 377.1, 387, 404.1, 415.3, 417.3, 431.1, 441.1, 469.1, 529.5, 551.1, 552.4, 553.3, 581.8, 633, 652.6, 683.3, 685.2, 717.1, 745, 880, 961, 1003, 1043

## Data Availability

Data is contained within the article.

## References

[1] Ramsing R., Santo R., Kim B.F., Altema-Johnson D., Wooden A., Chang K.B., Semba R.D., Love D.C. (2023). Dairy and plant-based milks: Implications for nutrition and planetary health. Curr. Environ. Health Rep..

[2] Blasi F., Pellegrino R.M., Alabed H.B., Ianni F., Emiliani C., Cossignani L. (2023). Lipidomics of coconut, almond and soybean milks-Comprehensive characterization of triacylglycerol class and comparison with bovine milk. Food Res. Int..

[3] Lagutin K., MacKenzie A., Bloor S., Scott D., Vyssotski M. (2022). HPLC-MS, GC and NMR profiling of bioactive lipids of human milk and milk of dairy animals (cow, sheep, goat, buffalo, camel, red deer). Separations.

[4] Clulow A.J., Salim M., Hawley A., Boyd B.J. (2018). A closer look at the behaviour of milk lipids during digestion. Chem. Phys. Lipids.

[5] Liu Z., Rochfort S., Cocks B. (2018). Milk lipidomics: What we know and what we don’t. Prog. Lipid Res..

[6] Sundekilde U.K., Larsen L.B., Bertram H.C. (2013). NMR-based milk metabolomics. Metabolites.

[7] John G.K., Mullin G.E. (2016). The gut microbiome and obesity. Curr. Oncol. Rep..

[8] Li Q., Zhao Y., Zhu D., Pang X., Liu Y., Frew R., Chen G. (2017). Lipidomics profiling of goat milk, soymilk and bovine milk by UPLC-Q-Exactive Orbitrap Mass Spectrometry. Food Chem..

[9] Kaur N., Chugh V., Gupta A.K. (2014). Essential fatty acids as functional components of foods-a review. J. Food Sci. Technol..

[10] Contarini G., Povolo M. (2013). Phospholipids in milk fat: Composition, biological and technological significance, and analytical strategies. Int. J. Mol. Sci..

[11] Righetti L., Rubert J., Galaverna G., Hurkova K., Dall’Asta C., Hajslova J., Stranska-Zachariasova M. (2018). A novel approach based on untargeted lipidomics reveals differences in the lipid pattern among durum and common wheat. Food Chem..

[12] Clish C.B. (2015). Metabolomics: An emerging but powerful tool for precision medicine. Mol. Case Stud..

[13] Matyash V., Liebisch G., Kurzchalia T.V., Shevchenko A., Schwudke D. (2008). Lipid extraction by methyl-tert-butyl ether for high-throughput lipidomics. J. Lipid Res..

[14] Fernando H., Kondraganti S., Bhopale K.K., Volk D.E., Neerathilingam M., Kaphalia B.S., Luxon B.A., Boor P.J., Shakeel Ansari G. (2010). 1H and 31P NMR Lipidome of Ethanol-Induced Fatty Liver. Alcohol. Clin. Exp. Res..

[15] Fernando H., Bhopale K.K., Kondraganti S., Kaphalia B.S., Ansari G.S. (2011). Lipidomic changes in rat liver after long-term exposure to ethanol. Toxicol. Appl. Pharmacol..

[16] Fernando H., Bhopale K.K., Boor P.J., Ansari G.S., Kaphalia B.S. (2012). Hepatic lipid profiling of deer mice fed ethanol using 1H and 31P NMR spectroscopy: A dose-dependent subchronic study. Toxicol. Appl. Pharmacol..

[17] Castañar L. (2017). Pure shift 1H NMR: What is next?. Magn. Reson. Chem..

[18] Chong J., Wishart D.S., Xia J. (2019). Using MetaboAnalyst 4.0 for comprehensive and integrative metabolomics data analysis. Curr. Protoc. Bioinform..

[19] Chong J., Liu P., Zhou G., Xia J. (2020). Using MicrobiomeAnalyst for comprehensive statistical, functional, and meta-analysis of microbiome data. Nat. Protoc..

[20] Jain S., Sekhar A. (2022). Elucidating the mechanisms underlying protein conformational switching using NMR spectroscopy. J. Magn. Reson. Open.

[21] HUANG X.-M., Sheng-Tao M., Jun-Tao C., Pei L., Xiang-Ying Z., Zhi-Qiang Y. (2017). Simultaneous determination of multiple persistent halogenated compounds in human breast milk. Chin. J. Anal. Chem..

[22] Trenerry V.C., Akbaridoust G., Plozza T., Rochfort S., Wales W.J., Auldist M., Ajlouni S. (2013). Ultra-high-performance liquid chromatography–ion trap mass spectrometry characterisation of milk polar lipids from dairy cows fed different diets. Food Chem..

[23] Maev I.V., Samsonov A.A., Palgova L.K., Pavlov C.S., Vovk E.I., Shirokova E.N., Starostin K.M. (2020). Effectiveness of phosphatidylcholine in alleviating steatosis in patients with non-alcoholic fatty liver disease and cardiometabolic comorbidities (MANPOWER study). BMJ Open Gastroenterol..

[24] Moore S., Costa A., Pozza M., Vamerali T., Niero G., Censi S., De Marchi M. (2023). How animal milk and plant-based alternatives diverge in terms of fatty acid, amino acid, and mineral composition. Npj Sci. Food.

[25] Vargas-Bello-Pérez E., Toro-Mujica P., Enriquez-Hidalgo D., Fellenberg M.A., Gómez-Cortés P. (2017). Discrimination between retail bovine milks with different fat contents using chemometrics and fatty acid profiling. J. Dairy Sci..

[26] Wang L., Li X., Liu L., da Zhang H., Zhang Y., Chang Y.H., Zhu Q.P. (2020). Comparative lipidomics analysis of human, bovine and caprine milk by UHPLC-Q-TOF-MS. Food Chem..

[27] Liu Z., Li C., Pryce J., Rochfort S. (2020). Comprehensive characterization of bovine milk lipids: Triglycerides. ACS Omega.

[28] Lindmark Månsson H. (2008). Fatty acids in bovine milk fat. Food Nutr. Res..

[29] Cossignani L., Giua L., Urbani E., Simonetti M.S., Blasi F. (2014). Fatty acid composition and CLA content in goat milk and cheese samples from Umbrian market. Eur. Food Res. Technol..

[30] Martínez-Padilla E., Li K., Blok Frandsen H., Skejovic Joehnke M., Vargas-Bello-Pérez E., Lykke Petersen I. (2020). In vitro protein digestibility and fatty acid profile of commercial plant-based milk alternatives. Foods.

[31] Ekanayaka R., Ekanayaka N., Perera B., De Silva P. (2013). Impact of a traditional dietary supplement with coconut milk and soya milk on the lipid profile in normal free living subjects. J. Nutr. Metab..

[32] Peñalvo J.L., Castilho M.C., Silveira M.I.N., Matallana M.C., Torija M.E. (2004). Fatty acid profile of traditional soymilk. Eur. Food Res. Technol..

[33] Oyeyinka A.T., Odukoya J.O., Adebayo Y.S. (2019). Nutritional composition and consumer acceptability of cheese analog from soy and cashew nut milk. J. Food Process. Preserv..

[34] Rico R., Bulló M., Salas-Salvadó J. (2016). Nutritional composition of raw fresh cashew (Anacardium occidentale L.) kernels from different origin. Food Sci. Nutr..

[35] Amores G., Virto M. (2019). Total and free fatty acids analysis in milk and dairy fat. Separations.

[36] Rund K.M., Carpanedo L., Lauterbach R., Wermund T., West A.L., Wende L.M., Calder P.C., Schebb N.H. (2024). LC-ESI-HRMS—Lipidomics of phospholipids: Characterization of extraction, chromatography and detection parameters. Anal. Bioanal. Chem..

[37] Castro-Gómez M., Rodriguez-Alcalá L.M., Calvo M.V., Romero J., Mendiola J., Ibáñez E., Fontecha J. (2014). Total milk fat extraction and quantification of polar and neutral lipids of cow, goat, and ewe milk by using a pressurized liquid system and chromatographic techniques. J. Dairy Sci..

[38] Welty F.K. (2020). Dietary treatment to lower cholesterol and triglyceride and reduce cardiovascular risk. Curr. Opin. Lipidol..

[39] Packard C.J., Boren J., Taskinen M.R. (2020). Causes and Consequences of Hypertriglyceridemia. Front. Endocrinol..

[40] Liu Z., Rochfort S. (2023). Lipidomics in milk: Recent advances and developments. Curr. Opin. Food Sci..

[41] McClain C.J., Barve S., Deaciuc I. (2007). Good fat/bad fat. Hepatology.

[42] Glick N.R., Fischer M.H. (2013). The role of essential fatty acids in human health. J. Evid.-Based Complement. Altern. Med..

[43] Jensen R.G., Ferris A.M., Lammi-Keefe C.J. (1991). The composition of milk fat. J. Dairy Sci..

[44] Yu E., Hu F.B. (2018). Dairy products, dairy fatty acids, and the prevention of cardiometabolic disease: A review of recent evidence. Curr. Atheroscler. Rep..

[45] Fernandes G.D., Gómez-Coca R.B., Pérez-Camino M.d.C., Moreda W., Barrera-Arellano D. (2017). Chemical characterization of major and minor compounds of nut oils: Almond, hazelnut, and pecan nut. J. Chem..

[46] Griffin L., Dean L. (2017). Nutrient composition of raw, dry-roasted, and skin-on cashew nuts. J. Food Res..

[47] Osipova D., Kokoreva K., Lazebnik L., Golovanova E., Pavlov C., Dukhanin A., Orlova S., Starostin K. (2022). Regression of Liver Steatosis Following Phosphatidylcholine Administration: A Review of Molecular and Metabolic Pathways Involved. Front Pharmacol..

[48] Ortega-Anaya J., Jiménez-Flores R. (2019). Symposium review: The relevance of bovine milk phospholipids in human nutrition-Evidence of the effect on infant gut and brain development. J. Dairy Sci..

[49] Muhamad Kamil N.A.I., Wan Ismail W.Z., Ismail I., Jamaludin J., Hanasil N.S., Ibrahim R.K.R. (2021). Analysis of milk from different sources based on light propagation and random laser properties. Photonics.

[50] Sharma N., Yeasmen N., Dube L., Orsat V. (2024). A review on current scenario and key challenges of plant-based functional beverages. Food Biosci..

[51] Jesús-José D., Hidalgo-Fuentes B., Rosas-Espejel M., Rayas-Amor A.A., Jiménez-Guzmán J., Zambrano-Zaragoza M.L., González-Reza R.M., Liceaga A.M., Aguilar-Toalá J.E. (2024). Antioxidant properties of soy-dairy milk blends fermented with probiotics. Agro Product..

